# Automated periodontitis diagnosis and staging using an end-to-end deep learning model on panoramic dental radiographs

**DOI:** 10.1007/s11282-026-00921-x

**Published:** 2026-04-17

**Authors:** My Huong Le, Xuan Hao Mai, Batzaya Tumur-Ulzii, So-Hyun Kim, Nam-Sik Oh

**Affiliations:** 1 Department of Dentistry, School of Medicine, Inha University, 366 Seohae-Daero, Jung-Gu, Incheon, Republic of Korea; 2 Gene Solutions, Ho Chi Minh City, Vietnam

**Keywords:** YOLOv11, Tooth segmentation, Keypoint detection, Periodontitis diagnosis, Radiographic bone loss, Panoramic radiographs

## Abstract

**Objectives:**

This study aimed to develop and validate a comprehensive deep learning model utilizing YOLOv11 for the automatic segmentation of teeth, detection of anatomical landmarks, and staging of periodontitis through the analysis of panoramic radiographs.

**Methods:**

A total of 607 panoramic radiographs were annotated by qualified dentists The YOLOv11-based system executed tooth segmentation and classified teeth into four anatomical categories, subsequently identifying six critical landmarks to facilitate the calculation of radiographic bone loss. The performance of the model was assessed using metrics such as precision, recall, F1-score, and mean average precision (mAP). Agreement with the assessments provided by dentists was evaluated using Pearson correlation coefficients (PCC) and intraclass correlation coefficients (ICC).

**Results:**

The segmentation model demonstrated exceptional performance, achieving a precision of 0.981, recall of 0.984, F1-score of 0.983, and an mAP50 of 0.994. The keypoint detection models also exhibited robust performance, with precision and recall exceeding 0.951 and mAP50 surpassing 0.958. Correlation and agreement with the assessments of dental professionals were highest for incisors (PCC = 0.877; ICC = 0.871) and lowest for canines (PCC = 0.712; ICC = 0.705) (*p* < 0.001).

**Conclusions:**

The proposed YOLOv11-based framework facilitates automated, high-precision tooth segmentation, landmark detection, and periodontitis staging, thereby providing a reliable clinical decision support tool for the diagnosis of periodontal disease.

**Supplementary Information:**

The online version contains supplementary material available at 10.1007/s11282-026-00921-x.

## Introduction

Periodontitis is a chronic inflammatory disease characterized by the irreversible destruction of the supporting structures of the teeth, including the gingiva, periodontal ligament, cementum, and alveolar bone [1]. Its insidious progression often remains undetected until it advances to a severe stage, ultimately resulting in tooth loss. Such outcomes negatively impact masticatory efficiency and facial aesthetics, thereby significantly affecting quality of life [2]. Once ranked as the sixth most prevalent chronic disease globally, the prevalence of periodontitis has escalated from 11.2% to approximately 19%, as reported by the World Health Organization. This increase underscores the urgent necessity for early diagnosis and timely intervention to avert permanent damage and enhance patient outcomes [3].

The clinical diagnosis of periodontitis has traditionally relied on the measurement of clinical attachment loss (CAL) utilizing a periodontal probe. However, diagnostic accuracy can fluctuate due to various factors, including probe thickness, angulation and positioning, the pressure applied, and the operator’s technical proficiency [4]. In instances where CAL measurement proves challenging, radiographic bone loss (RBL) is frequently utilized as an alternative diagnostic marker [1]. Several radiographic modalities – such as bitewing, periapical, and panoramic imaging –facilitate the detection of periodontitis with relatively low radiation exposure [5].

Conventional bitewing radiographs assess the distance between the cemento-enamel junction (CEJ) and the crestal bone; however, they provide limited visualization in cases involving deep or irregular bone defects [5]. Periapical radiographs offer a two-dimensional perspective from crown to root but necessitate multiple exposures for a comprehensive periodontal evaluation. Panoramic radiographs, endorsed by the American Dental Association for patients with generalized periodontal disease, deliver an efficient, low-dose, and rapid overview of the entire dentition [6]. These advantages render panoramic imaging the preferred modality in clinical environments [5]. Nevertheless, the manual interpretation of panoramic images is time-consuming and subject to inter- and intra-examiner variability, which can result in inconsistent diagnostic outcomes [7]. This limitation accentuates the need for automated diagnostic tools capable of enhancing both accuracy and efficiency in periodontitis detection.

Recent advancements in artificial intelligence (AI), particularly in deep learning (DL) techniques and convolutional neural networks (CNNs), have substantially transformed the analysis of medical and dental imaging. CNNs utilize multiple hierarchical layers to extract and synthesize image features, enabling efficient image recognition, segmentation, localization, and classification [8]. In the field of medicine, CNNs have exhibited remarkable efficacy in applications such as prostate cancer detection [9], classification of benign and malignant parotid tumors [10], and management of atherosclerosis [11]. In dentistry, they are increasingly employed to analyze annotated dental radiographs for the automated detection of oral pathologies, including dental caries, periapical lesions, periodontitis, and maxillary sinusitis [7, 12–15].

Among CNN-based models, You Only Look Once (YOLO) has emerged as a leading real-time object detection algorithm since its inception in 2015. By integrating region proposal and classification within a single neural network, YOLO facilitates end-to-end learning while significantly reducing computational time [16]. Its grid-based architecture predicts bounding boxes and class probabilities for each cell, thereby enabling rapid and robust image analysis. Due to these advantages, YOLO has been extensively applied to the detection of periodontitis using panoramic radiographs [7, 17, 18]. However, earlier versions of YOLO encountered difficulties in detecting small or densely clustered objects, which limited diagnostic precision.

To mitigate these limitations, YOLOv11 introduces two significant innovations: the Cross Stage Partial (C3k2) block with a kernel size of 2 and the Convolutional block with Parallel Spatial Attention (C2PSA) module. These components enhance computational efficiency and feature extraction, thereby facilitating more accurate detection of small and occluded structures [19].

This study proposes a two-stage, end-to-end automated diagnostic system for periodontitis utilizing YOLOv11. In the initial stage, the model exploits YOLOv11’s advanced instance segmentation capabilities to segment and classify individual teeth into four anatomical categories from panoramic radiographs. In the subsequent stage, six anatomical landmarks are detected to automatically calculate RBL percentages. Based on these measurements, periodontitis is staged in accordance with the 2017 classification guidelines set forth by the American Academy of Periodontology and the European Federation of Periodontology. By integrating real-time detection with clinically relevant staging, this system aims to facilitate early diagnosis, thereby preventing severe disease progression and its complications.

## Materials and methods

### Dataset

This study received ethical approval from the Inha University Institutional Review Board and the Inha University Data Review Board (IRB No. 2024–08-034; DRB No. DRB-2024–10-001). All panoramic radiographs were acquired at the Dentistry Centre of Inha University Hospital utilizing a dental panoramic X-ray machine (ORTHOPHOS XG 5 & CEPH, Dentsply Sirona, Bensheim, Germany). A total of 607 radiographic images were collected between 2021 and 2023. Images from patients exhibiting primary or mixed dentition, as well as those affected by significant image distortion, were excluded from the dataset to ensure consistency and reliability in the subsequent model training process. All patient data were fully anonymized prior to analysis in accordance with institutional data protection protocols and international ethical standards, thereby safeguarding the confidentiality of personal information throughout the study.

### Annotation and image processing

All images were annotated utilizing the Computer Vision Annotation Tool (CVAT), a platform extensively employed in medical image analysis for precise manual labeling. The annotation process was conducted independently by two dentists possessing over five years of clinical experience in diagnosing periodontitis. Any discrepancies between their annotations were reviewed and reconciled through consensus with a senior dentist who provided the final verification. This multi-layered validation process ensured a high degree of consistency and minimized subjective variability within the dataset.

The annotation protocol was specifically designed to support a two-stage deep learning architecture based on YOLOv11. In the first stage, each tooth in the panoramic radiograph was segmented and classified into one of four anatomical categories: incisor, canine, premolar, and molar. This segmentation facilitated the model’s capability to isolate and analyze each tooth individually, providing a standardized input for subsequent landmark detection. In the second stage, the model was trained to detect six anatomical keypoints per tooth (m1–d3), corresponding to the mesial and distal CEJ, alveolar crest levels, and root apex. For single-rooted teeth, the mesial and distal apex (m3 and d3) were annotated as a singular point due to their anatomical overlap. These six key anatomical landmarks, summarized in Table 1, were selected based on their clinical significance for measuring radiographic bone loss.

**Table 1:** Description of six anatomical key points used for keypoint detection

Point name	Meaning
m1	Mesial CEJ
m2	Mesial alveolar crest bone
m3	Mesial root apex
d1	Distal CEJ
d2	Distal alveolar crest bone
d3	Distal root apex

*CEJ* cemento-enamel junction

Following tooth segmentation and classification, each tooth was cropped and annotated with the corresponding key landmarks. Given that certain keypoints (particularly m1, m3, d1, and d3) were frequently located near the image borders, a 30-pixel white margin was incorporated around the cropped images to mitigate the risk of misinterpretation during both training and inference. This preprocessing step enhanced the stability and accuracy of keypoint localization by ensuring that no landmark was truncated or positioned too close to the image edge, which could otherwise diminish detection precision.

### Periodontitis staging standard

The staging of periodontitis was determined based on RBL measurements, calculated by selecting the higher bone loss percentage between the mesial and distal aspects of each tooth. This methodology adhered to the approach outlined by Jiang et al. (2022) and complied with the 2017 Consensus Classification jointly issued by the American Academy of Periodontology and the European Federation of Periodontology. Unerupted or abnormally positioned third molars were excluded from analysis due to their inconsistent bone morphology and limited clinical relevance to generalized periodontitis staging.

Teeth exhibiting alveolar bone loss of less than 2 mm from the CEJ were not clinically diagnosed as having bone resorption. Nonetheless, given the technical challenges associated with accurately measuring 2 mm on panoramic radiographs, this study did not differentiate between teeth without bone loss and those classified as Stage I. The staging criteria and clinical features utilized in this study are summarized in Table 2. This standardization ensured that the model’s staging predictions were clinically meaningful and directly aligned with widely accepted diagnostic guidelines.

**Table 2:** Staging criteria for periodontitis based on radiographic bone loss (RBL) and associated clinical features

Stage	Bone loss (%)	Typical features
I	< 15%	Early bone loss, usually no mobility or tooth loss
II	15–33%	Moderate bone loss in the coronal third, often associated with deeper probing and possible furcation involvement
III/IV	> 33%	Severe bone loss, may reach the middle third of the root, often associated with tooth mobility, vertical bone defects, and risk of tooth loss

### Model training procedure

All experiments were conducted on Google Colab utilizing an NVIDIA L4 GPU. Transfer learning was employed to enhance model convergence and improve performance, particularly given the relatively limited dataset size. Training proceeded for a maximum of 200 epochs, commencing with an initial learning rate of 0.001. An early stopping strategy, characterized by a patience parameter of 30 epochs, was implemented to mitigate overfitting; this ensured that training was terminated once performance on the validation set ceased to exhibit improvement. The weights of the best-performing model were automatically preserved for subsequent evaluation.

To retain anatomical details, the segmentation model was trained on images resized to 1280 × 1280 pixels, while the keypoint detection model utilized a resolution of 640 × 640 pixels to achieve a balance between accuracy and computational efficiency. The entire dataset was randomly partitioned into training, validation, and test sets in an 8:1:1 ratio. The training set was designated for model fitting and parameter optimization, the validation set was employed to fine-tune the model and alleviate overfitting, and the test set provided an unbiased assessment of model performance on previously unseen data.

**Fig. 1:**
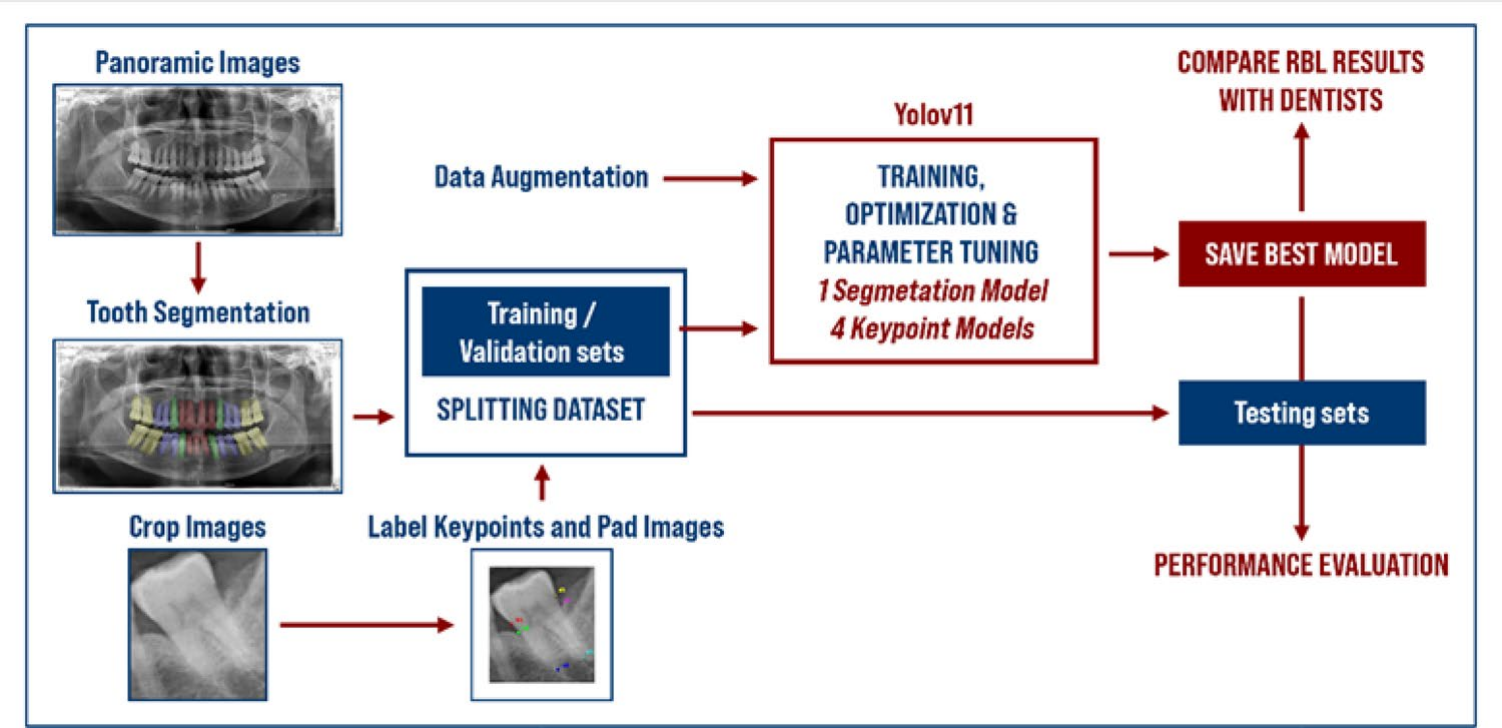
Workflow of tooth segmentation and anatomical keypoint detection using YOLOv11

Data augmentation techniques were implemented to address class imbalance and enhance the model’s generalizability. These techniques included brightness adjustment, random rotation of up to ± 10°, multiscale resizing, mosaic augmentation, and copy-paste augmentation. Such methodologies introduced controlled variability into the training data, thereby enabling the model to learn robust features and maintain accuracy across diverse imaging conditions. The overall workflow consisted of two primary stages. Initially, the segmentation model isolated and classified individual teeth from panoramic radiographs. Subsequently, four keypoint detection models were applied to the segmented regions to identify anatomical landmarks necessary for calculating radiographic bone loss and staging periodontitis. The performance of the final optimized model was subsequently compared with clinical assessments conducted by experienced dentists on an independent test set (Fig. 1).

### Evaluation metrics

The performance of the segmentation and keypoint detection models was evaluated using multiple standard metrics to provide a comprehensive assessment of predictive accuracy and robustness. For segmentation, the training process was monitored through loss curves, where a consistently decreasing loss over epochs indicated successful learning and optimization. A confusion matrix was generated to visualize classification performance across various tooth types, facilitating the identification of potential misclassification patterns. Precision was calculated to ascertain the proportion of correct positive predictions, while recall quantified the model’s ability to identify true positive cases. The F1-score, defined as the harmonic mean of precision and recall, served as a balanced measure of the model’s classification accuracy.

The degree of spatial overlap between predicted and ground-truth tooth boundaries was quantified using Intersection over Union (IoU), also referred to as the Jaccard index. Additionally, mean average precision (mAP) was computed to summarize the model’s segmentation and localization performance across all tooth classes. Evaluating mAP at multiple IoU thresholds allowed for the assessment of model reliability under both lenient and stringent conditions, thereby providing a more robust indication of performance stability.

For keypoint detection, analogous evaluation metrics were applied, supplemented by Object Keypoint Similarity (OKS), which measures the spatial correspondence between predicted and ground-truth keypoints. OKS functions similarly to IoU but focuses on landmark localization rather than segmentation boundaries. High OKS values indicated accurate localization of clinically relevant anatomical points, which is essential for reliable measurement of radiographic bone loss.

### Comparative evaluation with dentists

To establish a reference standard and assess clinical validity, three dentists with over five years of clinical experience at the Dentistry Centre, Inha University Hospital, independently conducted periodontitis staging and grading on the test dataset. These clinicians were blinded to the model’s predictions to mitigate bias and ensure an objective comparison between human and AI-based assessments.

The agreement between the model’s outputs and the evaluations provided by the dentists was assessed using two complementary statistical methods. The Pearson correlation coefficient (PCC) was calculated to determine the strength and direction of the linear relationship between the model’s predictions and the reference standard. Additionally, the intraclass correlation coefficient (ICC) was employed to evaluate the consistency and agreement between raters, specifically between the dentists and the model. All statistical analyses were conducted using IBM SPSS Statistics (Version 26.0, Armonk, NY, USA) and Python 3.9.13. A statistical significance threshold of *p* < 0.05 was established, and 95% confidence intervals were reported where applicable. These methodological approaches ensured that the model’s diagnostic performance was not only technically robust but also clinically comparable to expert human judgment.

## Results

### Dataset information

A total of 15,605 cropped tooth images were extracted from 607 panoramic radiographs. Of these images, 26.80% were classified as stage I periodontitis, 57.55% as stage II, and 15.65% as stage III or IV, in accordance with the 2017 Consensus Classification. The dataset was randomly partitioned into three subsets: a training set comprising 12,485 images, a validation set consisting of 1,560 images, and a test set containing 1,560 images (see Table 3). This stratified distribution ensured a balanced representation of disease stages across all subsets, thereby providing a stable foundation for model development and performance evaluation.

**Table 3:** Distribution of tooth samples across periodontal classification

Tooth type	Stage I	Stage II	Stage III/IV	Total
Incisor	710	2836	1109	4655
Canine	970	1299	111	2380
Premolar	1674	2475	308	4457
Molar	829	2371	913	4113
Total	4183	8981	2441	15,605

### Tooth segmentation model performance

**Fig. 2:**
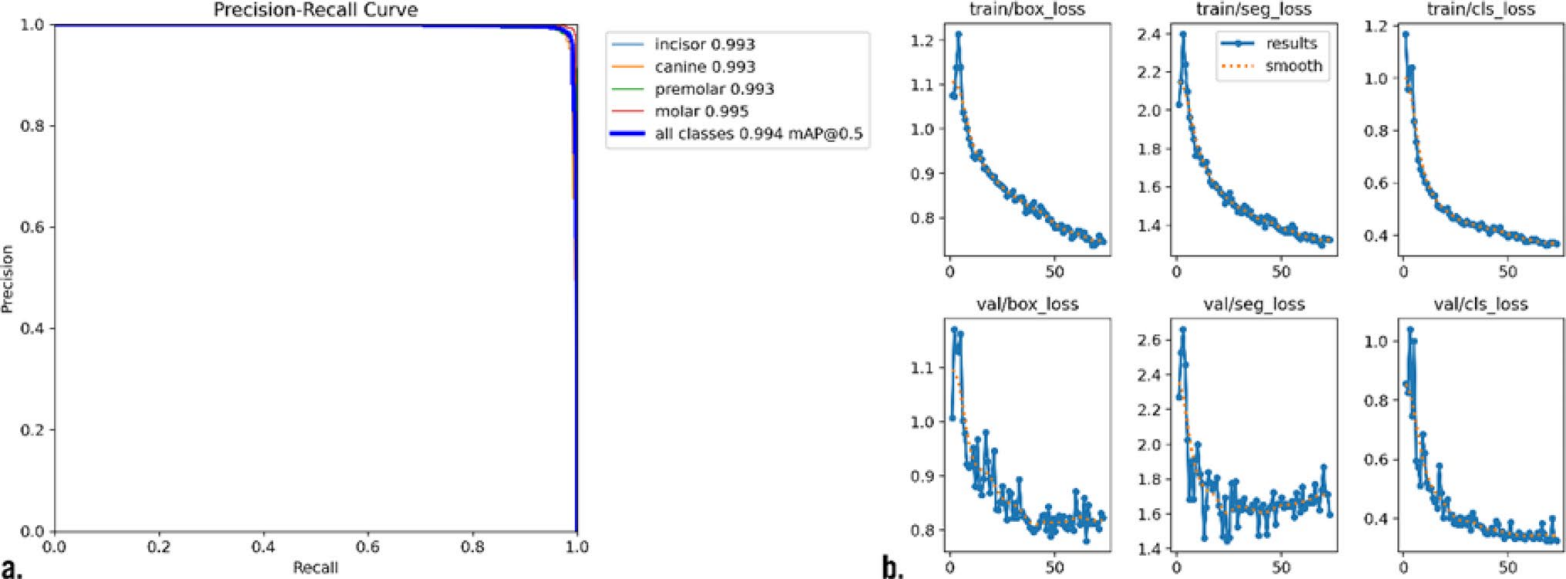
Precision-recall curve and loss curves in the training process of the segmentation model. **a**. Precision-recall curve, **b**. Loss curves

The performance of the YOLOv11 segmentation model exhibited exceptional accuracy in the detection and classification of individual teeth. The precision–recall curve depicted in Fig. 2a illustrates the model’s robust detection capability, with a mean average precision (mAP) of 0.994 across all tooth categories. This indicates a high proportion of accurately identified true positives with minimal false detections. As illustrated in Fig. 2b, the training and validation loss curves for both segmentation and classification demonstrated a consistent downward trend. The minimal and stable gap between the curves indicates effective model optimization, good generalization, and the absence of overfitting, thereby confirming the model’s robustness on unseen data.

**Fig. 3:**
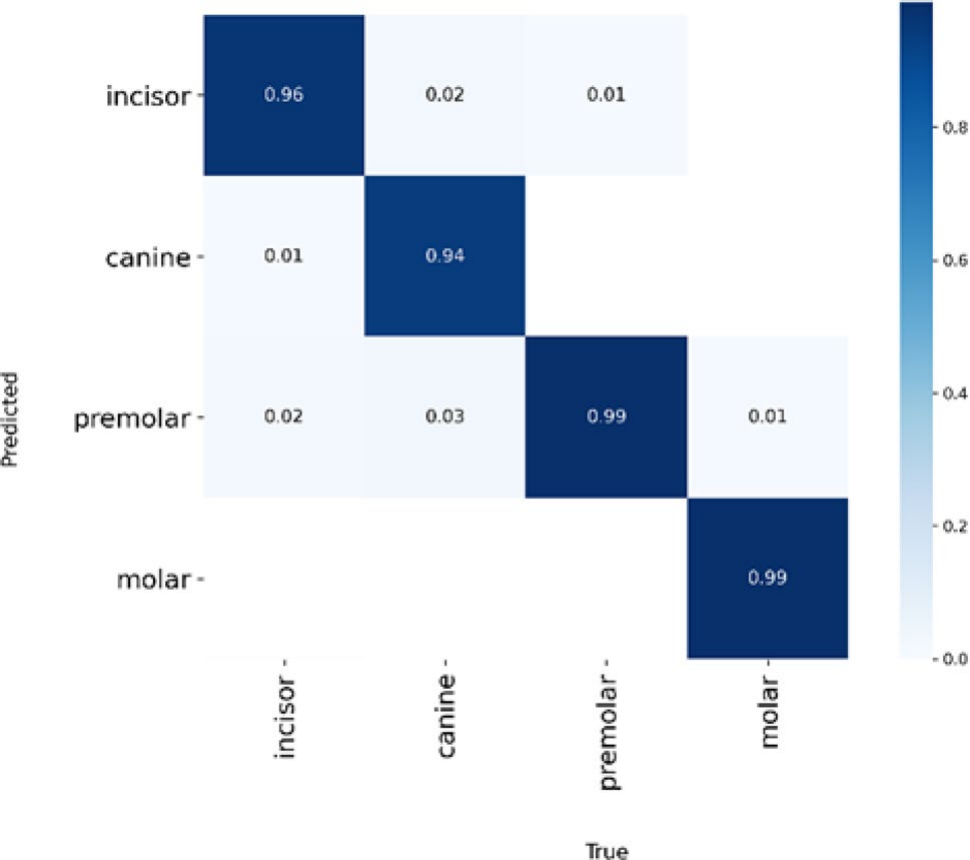
Confusion matrix of Yolov11 segmentation model for tooth classification on the test dataset

Figure 3 presents the confusion matrix that illustrates the classification accuracy of the YOLOv11 model across the four anatomical tooth categories. The model achieved classification accuracies of 96% for incisors, 94% for canines, 99% for premolars, and 99% for molars. Minor misclassifications were observed primarily between incisors and canines, attributable to their anatomical similarity and occasional overlapping features on panoramic radiographs. These results demonstrate the model’s capacity to reliably differentiate between similar tooth types, even under challenging imaging conditions.

Quantitative performance metrics are summarized in Table 4. The model achieved consistently high mAP values at an Intersection over Union (IoU) threshold of 0.5 (mAP50) across all tooth categories, ranging from 0.993 to 0.995. At a stricter IoU of 0.75, performance remained robust, with values exceeding 0.9. A marked decline in mAP was observed at IoU values above 0.85, indicating the increased challenge of maintaining perfect overlap at exceedingly high thresholds. This accounts for the overall mAP (0.5–0.95) range of 0.66 to 0.74 (see Table SI 1). Nonetheless, these values affirm that the segmentation model performs strongly across clinically relevant detection thresholds.

**Table 4:** Performance of the segmentation model across different tooth types

Tooth type	Precision	Recall	F1-score	mAP50	mAP
Incisor	0.97419	0.98721	0.98066	0.99333	0.67286
Canine	0.99154	0.97276	0.98206	0.99291	0.66427
Premolar	0.96697	0.98856	0.97764	0.99320	0.68721
Molar	0.99318	0.98628	0.98972	0.99475	0.74402
Total	0.98147	0.98370	0.98252	0.99355	0.69209

*mAP* mean average precision at Intersection over Union (IoU) thresholds from 0.50 to 0.95 using the Object Keypoint Similarity (OKS) technique, *mAP50* mAP at IoU threshold of 0.50

### Keypoint detection model performance

**Fig. 4:**
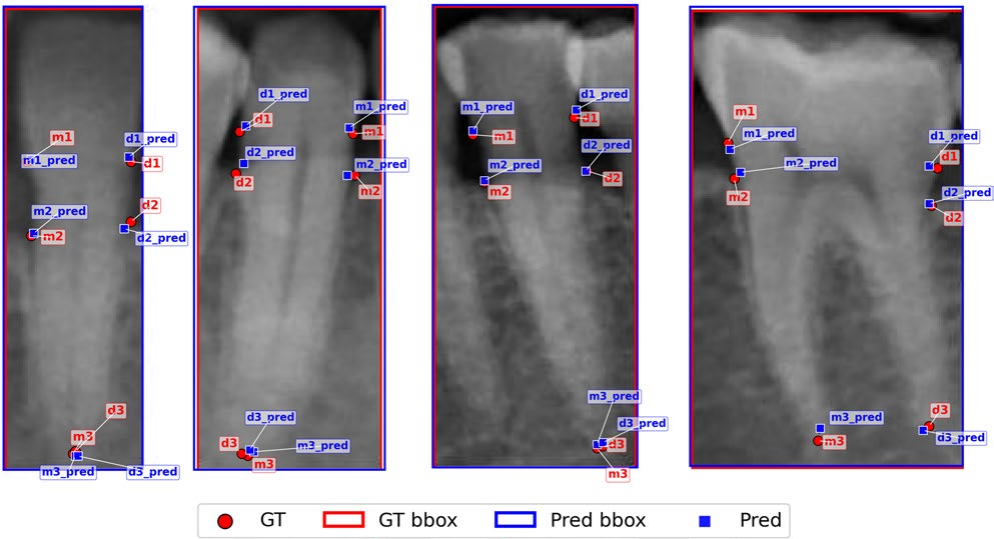
Example of keypoint annotations between the ground truth (dentist’s labeling) and model prediction. *GT* ground truth annotations by dentists; *GT bbox* the bounding box of the tooth defined by dentists; *Pred bbox* the bounding box generated by the model; *Pred* the model’s prediction

To evaluate the accuracy of anatomical landmark localization, separate keypoint detection models were trained for each tooth category using dentist-annotated ground truth as a reference. Figure 4 provides a qualitative comparison of dentist annotations and model predictions, illustrating a close alignment between the predicted and reference keypoint positions across various tooth types.

Table 5 presents the quantitative results for keypoint detection, stratified by tooth category. The model achieved excellent performance across all classes, with precision, recall, and F1-scores exceeding 0.951. The mean average precision at IoU 0.5 (mAP50) surpassed 0.958 across all tooth categories, and the mAP over the IoU range of 0.5–0.95 remained high (0.89–0.95), demonstrating the model’s ability to accurately localize anatomical landmarks even under stricter evaluation conditions (see Table SI 2). These findings confirm the stability of the keypoint models and their efficacy in generalizing across diverse tooth morphologies.

**Table 5:** Performance of keypoint detection models across tooth types for six key points

Tooth type	Precision	Recall	F1-score	mAP50	mAP
Incisor	0.96341	0.96352	0.96346	0.96369	0.89001
Canine	0.96616	0.96639	0.96627	0.95844	0.92355
Premolar	0.98194	0.98206	0.98200	0.99500	0.95399
Molar	0.95133	0.95146	0.95140	0.97694	0.93335

*mAP* mean average precision at Intersection over Union (IoU) thresholds from 0.50 to 0.95 using the Object Keypoint Similarity (OKS) technique, *mAP50* mAP at IoU threshold of 0.50

### Agreement between the model and dentists

The concordance between the model and dental practitioners regarding RBL measurement was assessed utilizing both the Pearson correlation coefficient (PCC) and the intraclass correlation coefficient (ICC). Table 6 presents the PCC values, which indicate a robust positive correlation between the model’s RBL measurements and those obtained by the dentists across all four dental categories. The most pronounced correlation was observed in incisors (PCC = 0.877), followed by molars (PCC = 0.842), premolars (PCC = 0.839), and canines (PCC = 0.712). All correlations were statistically significant, with p-values less than 0.001. Furthermore, Table SI 4 illustrates consistently high PCC values among the three dentists (*p* < 0.001), thereby confirming the substantial reliability and internal consistency of the clinical reference standard.

**Table 6:** Mean Pearson correlation coefficients (PCCs) with 95% confidence intervals for model and dentist measurements of radiographic bone loss

Tooth classification	Mean PCC
Incisor	0.877
Canine	0.712
Premolar	0.839
Molar	0.842

The ICC values further corroborate the strong agreement between the model and dental practitioners (Table 7). The ICC for incisors was the highest at 0.871, followed by molars at 0.841, premolars at 0.830, and canines at 0.705. All ICC values were statistically significant at *p* < 0.001. Table SI 5 reveals similarly elevated ICC values among the three dentists, indicating consistent clinical judgment and reinforcing the validity of the ground-truth annotations. These findings illustrate that the proposed automated system can yield RBL measurements that closely correspond with expert assessments, thereby underscoring its potential clinical utility in facilitating the staging of periodontitis.

**Table 7:** Mean intraclass correlation coefficients (ICCs) with 95% confidence intervals for model and dentist measurements of radiographic bone loss

Tooth classification	Mean ICC (model vs. dentists)	Mean ICC (among dentists)
Incisor	0.871	0.987
Canine	0.705	0.976
Premolar	0.830	0.973
Molar	0.841	0.987

## Discussion

Accurate diagnosis and staging of periodontitis are essential for timely intervention and effective treatment planning. However, conventional manual assessment of panoramic radiographs is often time-consuming, operator-dependent, and susceptible to inter- and intra-examiner variability. This subjectivity can result in inconsistent diagnoses and treatment outcomes, particularly in complex cases [1, 5, 7]. To address these limitations, recent studies have investigated deep learning models—particularly those based on YOLO—to develop automated and standardized periodontal diagnostic systems. Earlier frameworks typically required multiple models, such as U-Net or DeNTNet, to first segment individual teeth before applying YOLO for landmark detection [17, 20]. This multi-step process increased computational complexity and introduced numerous points of potential error. The present study utilizes the advanced capabilities of YOLOv11 to integrate instance segmentation and classification into a unified framework. This integration enables the model to classify teeth and detect key anatomical landmarks in a single, streamlined step, thereby reducing computational overhead while maintaining high diagnostic precision [21]. By reflecting the clinical workflow – first classifying teeth and subsequently detecting anatomical reference points – this approach replicates the decision-making process of dentists in a more objective and efficient manner.

Our segmentation model achieved precision, recall, and F1-scores exceeding 0.97 for all tooth categories, with mean Average Precision at 50% (mAP50) values greater than 0.99. These metrics are comparable to, and in some instances surpass, dentist-level performance. In comparison to previous YOLO variants, our YOLOv11 model outperformed YOLOv5 as reported by Beser (precision and F1-score of 0.98), YOLOv7 as reported by Muramatsu (precision and recall of 0.90), and YOLOv8 as reported by Xue (mAP50 of 0.94) [7, 22, 23]. At an Intersection over Union (IoU) of 0.75, our model maintained an mAP of 0.94, indicating robust localization accuracy even under more stringent evaluation criteria. The lower overall mAP (0.5–0.95) primarily reflects a decline in performance at extremely high IoU thresholds (> 0.90), which is anticipated in complex object detection tasks (Table SI 1). The combination of mAP50 and mAP75 offers a balanced perspective on model performance by capturing both sensitivity and localization precision [24]. Our mAP50 score of 0.994 and mAP75 score of 0.94 confirm the efficacy of YOLOv11 for automated dental image analysis.

Following segmentation, accurate identification of anatomical landmarks—specifically the CEJ, alveolar crest, and root apex—is critical for calculating RBL. To this end, we developed four keypoint models, one for each tooth type, that achieved precision, recall, and F1-scores exceeding 0.95. The mAP50 values were consistently above 0.95, and the models maintained strong performance at stricter Object Keypoint Similarity (OKS) thresholds (0.89–0.95). These results demonstrate that our keypoint models are capable of robust and precise landmark detection, which is a prerequisite for automated periodontal staging. In contrast, previous YOLO-based approaches exhibited significantly lower performance. For example, Li et al. 2025 reported lower scores using YOLOv8 (precision 0.76, recall 0.64, F1 0.68) [19], and Uzun et al. 2025reported similar figures using YOLOv5 (precision 0.75, recall 0.76, F1 0.76) [18, 25]. By leveraging YOLOv11’s pose estimation capabilities, our model significantly improves upon prior methods, ensuring accurate detection of the CEJ, alveolar crest, and root apex across diverse tooth morphologies.

The strength of our model is further supported by high concordance with clinical assessments. The mean intraclass correlation coefficient (ICC) values demonstrated strong agreement between model predictions and dentists’ measurements for incisors (0.871), molars (0.841), and premolars (0.830). Notably, our model outperformed Li’s framework for incisors (ICC = 0.78) and premolars (ICC = 0.76), underscoring its enhanced capacity to predict landmarks in anatomically complex or overlapping regions [18]. However, the ICC for canines was lower at 0.705. This reduction can be attributed to limited model performance in Stage III/IV canines, where recall and F1-scores were comparatively lower (Table SI 3). Advanced-stage canine periodontitis is relatively uncommon in clinical practice, resulting in underrepresentation in the dataset and, consequently, diminished model training effectiveness [26, 27].

These findings underscore a critical nuance in the application of deep learning models to panoramic dental imaging. Model performance may fluctuate based on factors such as tooth region, disease severity, and the intrinsic characteristics of the images. Panoramic radiography is widely recognized as an effective imaging modality for initial screening and treatment planning, providing extensive visualization of alveolar bone levels, patterns of bone loss, and furcation involvement [28–30]. Nonetheless, it possesses inherent limitations. Panoramic images may underestimate early bone loss, inadequately capture fine structural details, and are susceptible to geometric distortion and overlapping, particularly in the anterior and premolar regions [31–33]. Therefore, panoramic imaging should not serve as the sole basis for periodontal diagnosis. It is most efficacious when integrated with a comprehensive clinical examination and supplemented by periapical or bitewing radiographs in instances where higher spatial resolution is necessitated [28].

Subsequent research should prioritize the development of multimodal AI-based periodontal diagnostic systems that amalgamate panoramic radiography with higher-resolution imaging and clinical data. The integration of radiographic features with clinical parameters such as probing depth, CAL, and bleeding on probing could yield a more reliable and standardized diagnostic tool, enhancing both accuracy and clinical decision support. Furthermore, augmenting training datasets to encompass underrepresented tooth types and advanced disease stages will contribute to improving model generalizability and minimizing performance disparities across tooth categories.

In conclusion, this study successfully developed and validated an end-to-end system based on YOLOv11, designed to segment individual teeth and detect key anatomical landmarks within a single automated pipeline. By accurately measuring radiographic bone loss and staging periodontitis, the model attained performance levels that are comparable to those of experienced dental practitioners. Furthermore, the system exhibited strong concordance with expert clinical assessments, underscoring its potential utility as a diagnostic adjunct in dental practice. The implementation of this automated system has the potential to mitigate diagnostic variability, enhance workflow efficiency, and facilitate standardized periodontal assessments across diverse clinical settings. With additional integration of multimodal data and refinement for underrepresented tooth categories, such AI-driven methodologies present significant promise for advancing precision diagnosis and management of periodontitis in routine dental care.

## Supplementary Information

Below is the link to the electronic supplementary material.

Supplementary Material 1

## Data Availability

No datasets were generated or analysed during the current study.
