# Supplementary material for: Automated periodontitis diagnosis and staging using an end-to-end deep learning model on panoramic dental radiographs
Source: Oral Radiol. 2026 Apr 17;42(3):801–9. doi: 10.1007/s11282-026-00921-x (PMC13291031; doi:10.1007/s11282-026-00921-x)
Supplement: Supplementary file 1 — Supplementary Material 1 [file 11282_2026_921_MOESM1_ESM.pdf]

Supplementary Information (SI)

ORIGINAL ARTICLE

Automated periodontitis diagnosis and staging using an end-to-end deep learning model on panoramic dental radiographs

My Huong Le[1], Xuan Hao Mai[2], Batzaya Tumur-ulzii[1], So-Hyun Kim[1*], Nam-Sik Oh[1*]

[1]Department of Dentistry, School of Medicine, Inha University, Incheon, Republic of Korea

[2]The Master Program in Smart Healthcare Management, International College of Sustainability Innovations, National Taipei University, New Taipei, Taiwan

[*] **Corresponding author**:

So-Hyun Kim, D.D.S, M.S.D, Ph.D

Department of Dentistry, School of Medicine, Inha University,

366 Seohae-daero, Jung-gu, Incheon, Republic of Korea

Email: shkim19830405@inha.ac.kr

Tel: 032-890-2470; Fax: 032-890-2475

Nam-Sik Oh, D.D.S, M.S.D, Ph.D

Department of Dentistry, School of Medicine, Inha University,

366 Seohae-daero, Jung-gu, Incheon, Republic of Korea

E-mail: onsdo@inha.ac.kr

Tel: 032-890-2470; Fax: 032-890-2475

Table SI 1. Mean average precision (mAP) at different Intersection over Union (IoU) thresholds of the segmentation model across tooth types

IoU	Incisor	Canine	Premolar	Molar	Total
0.50	0.99333	0.99291	0.99320	0.99475	0.99355
0.55	0.99333	0.99291	0.99320	0.99475	0.99355
0.60	0.99333	0.98755	0.99320	0.99475	0.99221
0.65	0.98690	0.98755	0.99320	0.99475	0.99060
0.70	0.97693	0.97404	0.99320	0.99452	0.98467
0.75	0.90827	0.93284	0.95065	0.96391	0.93892
0.80	0.69265	0.58728	0.70491	0.85970	0.71114
0.85	0.17504	0.17976	0.24146	0.56800	0.29107
0.90	0.00886	0.00784	0.00906	0.07505	0.02521
0.95	0.00000	0.00000	0.00000	0.00000	0.00000
**0.50 – 0.95 (mean)**	0.67286	0.66427	0.68721	0.74402	0.69209

IoU Intersection over Union

Table SI 2. Mean average precision (mAP) at different Intersection over Union (IoU) thresholds of the keypoint detection models across tooth types

IoU (OKS)	Incisor	Canine	Premolar	Molar
0.50	0.96369	0.95844	0.97639	0.95513
0.55	0.94126	0.94298	0.94602	0.95513
0.60	0.92696	0.93831	0.93839	0.95513
0.65	0.92211	0.93131	0.93268	0.95215
0.70	0.92002	0.92919	0.92875	0.95215
0.75	0.90650	0.92919	0.92875	0.95052
0.80	0.89202	0.92919	0.92574	0.94778
0.85	0.87224	0.91363	0.91572	0.93792
0.90	0.82715	0.91185	0.90303	0.91289
0.95	0.72818	0.85136	0.82573	0.81472
**0.50 – 0.95 (mean)**	0.89001	0.92355	0.92212	0.93335

OKS Object Keypoint Similarity

Table SI 3. Performance of keypoint detection models for each periodontitis stage

Model	Metric	Stage I	Stage II	Stage III	Macro	Micro	Weighted
Incisor	Precision	0.76786	0.77812	0.80247	0.78281	0.78112	0.78235
	Recall	0.60563	0.90141	0.58559	0.69754	0.78112	0.78112
	F1-score	0.67717	0.83524	0.67708	0.72983	0.78112	0.77348
Canine	Precision	0.70940	0.80672	0.50000	0.67204	0.75630	0.75288
	Recall	0.85567	0.73846	0.09091	0.56168	0.75630	0.75630
	F1-score	0.77570	0.77108	0.15385	0.56688	0.75630	0.74444
Premolar	Precision	0.70395	0.71636	0.73684	0.71905	0.71300	0.71311
	Recall	0.63690	0.79757	0.45161	0.62870	0.71300	0.71300
	F1-score	0.66875	0.75479	0.56000	0.66118	0.71300	0.70884
Molar	Precision	0.67470	0.78175	0.79221	0.74955	0.76214	0.76252
	Recall	0.67470	0.83122	0.66304	0.72299	0.76214	0.76214
	F1-score	0.67470	0.80573	0.72189	0.73411	0.76214	0.76061

Table SI 4. Pearson correlation coefficients (PCCs) for model vs. dentist measurements of radiographic bone loss

**Tooth Type**	**Dentist 1**	**Dentist 2**	**Dentist 3**
Incisor	0.878	0.857	0.898
Canine	0.710	0.684	0.759
Premolar	0.853	0.801	0.865
Molar	0.836	0.816	0.872

95% confidence intervals, $p < 0.001$

Table SI 5. Intraclass correlation coefficients (ICCs) for model vs. dentist measurements of radiographic bone loss

Tooth Type	Dentist 1	Dentist 2	Dentist 3
Incisor	0.871	0.849	0.894
Canine	0.695	0.668	0.751
Premolar	0.843	0.788	0.858
Molar	0.836	0.816	0.870

95% confidence intervals, $p < 0.001$
